# Supplementary material for: Community Involvement in Onchocerciasis Post-elimination Surveillance in Bududa District, Eastern Uganda: A cross-sectional study
Source: PLoS Negl Trop Dis. 2024 Jul 16;18(7):e0012270. doi: 10.1371/journal.pntd.0012270 (PMC11251607; doi:10.1371/journal.pntd.0012270)
Supplement: Attachment — Submitted filename: Point by Point Response letter.docx [file pntd.0012270.s002.docx]

**Subject: Revised Manuscript Submission - "Community Involvement in Post-elimination Surveillance in Bududa District, Eastern Uganda: A Cross-Sectional Study" to Neglected Tropical Diseases**

Dear Matthew C Freeman,

I extend my sincere gratitude for affording me the opportunity to submit the revised draft of my manuscript, titled "Community Involvement in Post-elimination Surveillance in Bududa District, Eastern Uganda: A Cross-Sectional Study," to Neglected Tropical Diseases. I want to express my appreciation for the time and effort you and the reviewers have devoted to providing valuable feedback on my work. I am particularly thankful to the reviewers for their insightful comments, which have greatly contributed to enhancing the quality of my paper. I have diligently incorporated changes to address the majority of the suggestions made by the reviewers, and these modifications have been clearly marked within the manuscript.

I am thankful for your meticulous review of our manuscript. Enclosed is our detailed point-by-point response to each comment for your perusal.

| **Reviewer’s comment** | **Response to comment** | **Page numbers** |
| --- | --- | --- |
| Reviewer #1 | | |
| **Methods** |  |  |
| The authors stated the objective of the study. In the later part of the introduction, the authors wrote that ‘it is uncertain on the role and extent of the communities in Post elimination surveillance (PES). Also, it is not known whether the capacity created during Post Treatment Surveillance (PTS) period is sufficient to enable the communities to implement sound and vibrant PES activities. However, the authors did not mention the roles and functions of the communities in PES and the capacity created during PTS. I kindly suggest that the authors include this information to justify the reason for their study. | Thank you very much for this comment. It is true, we needed to highlight the roles and functions of communities during PES. The significance of PES is to closely monitor the sustainability of onchocerciasis elimination status. This is important as it provides data on the risk of onchocerciasis recrudescence to support WHO certification. This has been incorporated on page 1 with track changes | Page 1 |
| Authors have obtained permission from the appropriate ethical regulatory body for the study. Please let the ‘ethics approval and consent to participate’ statement appear at the methods section of the manuscript and not to the end. | Thank you very much for this comment. This has been moved to the methodology section of the manuscript. However, we had placed it at the end as it was guided by the journal format. | See page 10 |
| The authors clearly described the appropriate population suitable for the study. | Thank you very much for this comment. |  |
| The sample size was determined from sample size calculation and is sufficient to ensure adequate power to address the hypothesis being tested | Thank you very much for this comment. |  |
| The first sentence that begins paragraph 2 under the background of page 1 is not clear and understandable. I suggest that the authors re-write it with clarity to the readers. | Thank you very much for this comment. Indeed, the sentences were not clear. However, we have addressed this be re-writing it for clarity as recommended | See page 2 |
| The statement ‘were replicated in Africa’ that ends the sentence; Ivermectin treatment for at-risk populations, ----- of page 1 is not clear. | Thank you very much for this comment. This statement has been re-written for clarity. | See page 2 |
| Kindly delete one of the ‘in’ in the sentence in page 1 | This change has been affected as recommended. | See page 2 |
| Please this sentence ‘At the commencement of Uganda’s elimination efforts in 2007, a population of 4.9 million people from 16 foci excluding Victoria Nile focus is approximately 80 km from the source of River Nile near Jinja town extending to a point where the river became sluggish before reaching Lake Kyoga) faced the risk of onchocerciasis. However, as of August 2023, this number has dwindled to 697,032 people in the Country’ is too long and not clear. I suggest the authors break it into smaller sentences with clarity. The authors should also cite the source of the information. -       There should be a full stop sign before the beginning of this sentence: ‘By close of 2017, three countries -Republic of Venezuela, Uganda, and Sudan had already ceased mass drug administration (MDA) and completed three years of post-treatment surveillance in at least one transmission area’. I also suggest the authors cite the source of this information. | Thank you very much for this comment. The sentences in question have been re-written and full stop included as recommended.  Additionally, the suggestion to include citation has also been addressed by correctly citing the authors. | See page 2 |
| Please the first appearance of these words; ‘PES’ and ‘PTS’ should be written in full and the abbreviated forms in bracket after which the abbreviated forms alone can subsequently be used in the write-up. | Thank you so much for this comment. This recommendation has been adopted | Page 2 |
| Please this sentence: ‘As of 2023, the country still has 7 districts in PTS, while 5 border districts are under transmission interruption suspected (interventions still on-going)’ is not clear. I suggest the authors re-write it with clarity. | Thank you very much for this comment. This has been addressed for clarity as recommended | See page 2 |
| ‘Onchocerciasis’ is presented in the manuscript in/not in italic. I suggest it should not be italicized. | Thank you very much for this comment. This has been corrected throughout the document, except for the ones in qualitative quotes that must appear in italic. | See page 1&2 |
| **Results** |  |  |
| The results are not clear, the tables are not formatted well and cannot be understood. The authors also failed to indicate the statistical test they used to analyze their results. | Thank you for this comment. This has been included under every table footer. We used chi square test, fisher exact and logistic regression. | See page 11 |
| For instance, Table 3, 4, 5 contain variables that do not add up to the respective totals as indicated by the authors. The total percent computed is either less than or more than 100% | Thank you for this comment. The tables are now adding up, except for some places where we did multiple response tabulations and presented the percentage of cases. Additionally, we also presented column percentages mostly instead of row percentages, and this could explain why some tables might have numbers not adding up row wise. |  |
| Table 5 appeared before Table 4 and some of the tables are overlapping which is incorrect. | Thank you very much for this observation. It was a typo error which has been corrected | See page 13 |
| I suggest that the test statistics employed in the analysis of the results should be spelt out clearly under ‘Data analysis’ statement. I also suggest the statistical test used for the analysis of the results should be provided under each table. | Thank you for this comment. This has been incorporated | See page 8 and 12 |
| Please delete the statement ‘of data collection’ that appeared at the end of the sentence under ‘Study design’. | Thank you for this comment. This statement was deleted | See page 5 |
| The authors failed to provide citation to the statements made under ‘study setting’. I suggest that the authors cite any piece of information and ideas in the manuscript that are not theirs. | Thank you very much for this comment. This observation has been addressed by insertion of the relevant citations under the methodology section | See page 6 |
| The sentence ‘Most of the participants were the original inhabitants of the area reported at 66% as detailed in Table 2’ is not clear. Kindly present this with clarity. | Thank you for this observation. We have responded to this by re-writing it for clarity. | See page 11 |
| **Conclusion** |  |  |
| The authors provided conclusions which are supported by the data presented in the manuscript. The benefit of the findings to the advancement of knowledge on the topic under study have been discussed and public health relevance has also been touched on. However, the limitation of the study is not mentioned. I suggest that the authors provide information on the limitations of the study and this information should come before the conclusion statement. | Thank you very much for this comment. This was an omission done during manuscript drafting. Our original book had strength and limitation. However, this has now been brought to the manuscript as recommended | See Page 24 |
| **Editorial and Data Presentation Modifications** |  |  |
| I suggest the authors take into consideration the recommendations made in the methods and results section to improve on the manuscript. | Thank you. We have taken all the suggested measures here |  |
| **Summary and General Comments** |  |  |
| The authors did not provide page and line numbers making it difficult to provide review comments to specifics in the manuscript. | Thank you very much. The page numbers of have now be provided in the point by point response letter. |  |
| The manuscript contains incorrect sentence constructions. | The sentences have been re-written to portray clarity, in areas that were found wanting. |  |
| The ‘Author’s summary’ as required by the journal is missing in the manuscript. I suggest the authors provide this important information after the ‘Abstract’ statement. | Thank you very much for this great observation. This has been included in this manuscript on page 1, following the abstract | See page 1 |
| The authors have also not provided information on authors’ contributions. Kindly provide this information after the acknowledgement statement. | Thank you very much for this comment. However, this was provided in the submission system online during the initial submission. |  |
| Most of the ideas presented are not cited. I suggest that the authors provide in-text citations as well as references to piece of words or ideas that are not theirs. | Thank you very much for this comment. This suggestion has been taken care off with citations included in all areas recommended. |  |
| The format used to present the bibliographies is incorrect. I recommend that the authors edit the bibliography to suit the journal’s style. | Thank you very much for this comment. This formatting style has been adopted | See reference page |
| Reviewer #2 | | |
| **Method** |  |  |
| The description of the study settings in Bududa district is comprehensive and highlights the significant achievements in onchocerciasis prevention. To further enrich this section, it would be beneficial to include details about the pre-control level of endemicity (hypo-, meso-, or hyperendemicity) and the specific interventions or ecological changes that contributed to onchocerciasis elimination. Such information could greatly aid in understanding the context and implications for the success and interpretability of post-elimination surveillance (PES). I recognize there is already some, specific information regarding this in the sampling procedure for the two sub-counties sampled. | Thank you very much for this comment. Indeed, your input here is well adopted based on the need to create an understanding of the context. Therefore, we have addressed this comprehensively under settings. | See page 5 |
| The study population's description, including the exclusion criteria and sample size estimation, is well-articulated. The rationale behind selecting the two sub-counties for sampling, considering their proximity to blackfly breeding sites and high endemicity, is sound and justifiable | Thank you very much for this comment. |  |
| In terms of statistical analysis, the approach seems adequately described and appears to align with the study's objectives. Nevertheless, for a clearer understanding of the metrics used in the study, elaborating on what constitutes a "yes" in terms of community involvement in post-elimination surveillance would be useful. Although this is addressed later in the results and discussion, understanding the basis of these criteria—whether they stem from an open question or a predefined set of activities—would provide greater clarity to the reader. | Thank you very much for this comment. This has been addressed under data analysis section | See page 8 |
| Ultimately, the mixed-methods study designs contribute to the understanding of the topic in question and the ethical approval seems adequate. | Thank you very much sir for acknowledging this as the strength of our study. |  |
| Results |  |  |
| I was unable to locate any reference in Table 2 to the mentioned variable stating '66% of the participants were original inhabitants of the area.' | Thank you for this observation. This was just a misguided statement. It has been deleted. | See page 11 |
| The 'Knowledge and Awareness about Onchocerciasis Disease' section provides valuable insights into the local population's understanding of the disease and anecdotal evidence of the continued presence, albeit limited, of the 'Simulium' vector. This suggests a potential risk of the disease's resurgence. | Thank you very much for your line of thought here. In fact, this was also our initial assumption at the beginning, but we later discovered that, as much as the flies were still there, they were no longer carrying the vectors as reported by the vectors team. |  |
| Additionally, the 'Knowledge on Implementation of Onchocerciasis PES Activities' section is informative. It may be worthwhile to explore in the discussion the potential of epilepsy prevalence as an indicator of resurgent onchocerciasis transmission in PES, especially given its high association with onchocerciasis in regions of Uganda, South Sudan, and Cameroon (among other countries). | Yes, it is true; Eastern Uganda has the highest prevalence of epilepsy in Uganda, with a rate of 2.16% compared to other regions. However, there is also variation in this prevalence among age groups, with the majority being found in adults. Unfortunately, in this study, we did not extensively explore this coexistence during our literature review due to the limited research on this aspect in the Eastern region. This is why we did not discuss it in our discussion section. However, it would certainly be a valuable area to explore, given the high prevalence found in this part of the region by the School of Public Health, Makerere. |  |
| A particular sentence appears incomplete: 'The community was 14 times more likely to be engaged in PES activities [AOR = 14, 95% CI = 2.5-81.7].' Perhaps it could be concluded with a phrase like 'if aware of district support for onchocerciasis community activities.' | Thank you very much for this comment. This is true, and it might have been an error of omission. But it has been rectified | See page 15 |
| The qualitative analysis effectively highlights challenges in sustaining PES post-PTS, notably funding constraints, referral mechanisms, and motivation issues, alongside the selection of accessible communities, though the focus here should ideally be on a representative community sample." | Thank you for this comment. Yes, this is true and using the kish Leslie formula we included a large sample size of 422 participants. And using enumeration area concept of allocating sample, we tried to pick a representative sample per each community. | See page 6. |
| **Conclusion** |  |  |
| In the initial discussion paragraph, the categorization of individuals who observed blackflies in their vicinity as part of, or associated with, post-elimination surveillance (PES) warrants further discussion. How does this relate to the possibility of onchocerciasis resurgence in the area? If correlated with the possibly permanent vector disappearance (or reduction) in parts of Uganda, then it would be beneficial to mention so. | Thank you for highlighting this aspect. We appreciate your observation. In our interviews with entomological assistants, they acknowledged the presence of the flies but disputed claims of them having infectious bites any longer. This perspective led us to categorize individuals observing blackflies in their vicinity as part of post-elimination surveillance (PES). However, we understand the importance of further discussion on how this categorization relates to the possibility of onchocerciasis resurgence, especially in correlation with the potential permanent vector disappearance or reduction observed in other parts of Uganda. We will delve deeper into this aspect in our subsequent discussions to provide a more comprehensive understanding of the situation |  |
| With the limited literature available on PES, the study benefits from comparing its findings with those of similar diseases targeted for elimination and critically interpretes it. It's also important to note that the Bududa community received education and awareness campaigns during PTS, a detail that could have been mentioned before, perhaps within the methodology section (when describing the community). | Thank you for your insightful comment. We appreciate the suggestion to include information about education and awareness campaigns in the methodology section, particularly during the description of the community. However, it's important to note that the implementation of pre-treatment surveillance (PTS) activities posed challenges in obtaining precise details. Our reliance on information from the Uganda onchocerciasis guidelines 2012 and WHO 2016 guidelines, which emphasize awareness creation through community health education and sensitization, shaped our study design. While we couldn't be certain of the exact nature of education during PTS, we acknowledge the importance of this aspect and we have considered expanding on write up, to enhance the discussion on community education in our study. |  |
| The correlation between seeing blackflies and increased engagement in PES activities is intriguing. It raises the question of whether this observation is more indicative of heightened awareness rather than actual blackfly prevalence. Additional information on the presence or reduction of blackflies in the district would offer valuable context to the reader. | Thank you for highlighting this interesting point. We acknowledge that the correlation between seeing blackflies and increased engagement in PES activities might be indicative of heightened awareness rather than actual blackfly prevalence. While we recognize this as an unexpected finding from our analysis, we couldn't delve deeper into this aspect during the study. It could indeed be an avenue for future research or a valuable consideration for the program to explore further, potentially informing strategies for community engagement and awareness campaigns. We appreciate your insight, and your comment added depth to the interpretation of our results. |  |
| Editorial and Data Presentation Modifications |  |  |
| Title: I recommend including "onchocerciasis" or "Onchocerca volvulus" in the title to specify that the study focuses on the elimination of this specific parasite. | Thank you very much for this comment. It is very necessary as it depict prompt understanding of what the research is all about. | See title page |
| Abstract: The abstract is clearly structured, effectively outlining the study's introduction, aims, methodology, results, and conclusion. However, elaborating on the role and implications of community involvement in post-elimination surveillance in the background section would provide more depth. If space is a concern, consider shortening the first two lines of the abstract, which may be more appropriate for the introduction/background of the study. | Thank you very much for this comment. This is true and we have included examples of community roles in the background. | See page 1 |
| Background: 1) In the initial discussion of onchocerciasis clinical manifestations, it would be beneficial to include a mention of the strong association between onchocerciasis and neurohormonal conditions, specifically 'onchocerciasis-associated epilepsy.' This link has been explored in previous studies in Uganda and could be beneficial for the current research. | Thank you for this insightful comment. It has been incorporated. However, despite the high prevalence of epilepsy in our study setting in Eastern Uganda, nothing is known about the association between onchocerciasis and epilepsy in Bududa district. |  |
| 2) Regarding the sentence on the elimination of the disease in the Americas: 'However, by 2012, through the implementation of bi-annual Ivermectin treatment for at-risk populations, the disease was successfully eliminated in the Americas,' it would be informative to clarify that ivermectin was administered biannually and in some cases quarterly in the Americas. This detail could help explain the success in eliminating onchocerciasis in that region, in contrast with the challenges faced in some parts of Africa (more endemicity, effective vectors, logistical challenges in increasing CDTI frequency, etc.). | Thank you very much for this key suggestion. This has been implemented on page 2 | See page 2 |
| 3) Additionally, in the statement 'The African continent adopted a comparable strategy, using Ivermectin to treat affected communities, but complemented it with a vector control/elimination initiative,' it would be clearer to specify that only certain parts of Africa, particularly those within the Onchocerciasis Control Program in West Africa (OCP), implemented vector control measures. | Thank you for this great comment. We have adopted this as recommended | See page 2 |
| 4) The background section would also benefit from a detailed description of the journey from onchocerciasis control to elimination. This should include an explanation of the definitions of post-treatment surveillance (recommended for at least 3 years to achieve onchocerciasis elimination according to the 2016 WHO guidelines) and post-elimination surveillance, and how the latter is currently conducted. For further insights, I recommend reviewing the paper 'Onchocerciasis Elimination: Progress and Challenges' by Lakwo et al., especially figure 1. | Thank you very much for this comment. This has now been included under the background section | See page 4 |
| 5) Finally, while the abbreviation PES is fully explained in the abstract, it should also be explicitly defined as 'post-elimination surveillance' when first used in the main text (e.g. in 1 city have attained Onchocerciasis elimination status and are under PES [8].). | Thank you very much for this comment. This has been implemented has suggested here |  |
| Overall remark: The manuscript is overall understandable but could benefit from a comprehensive review to further enhance its linguistic clarity. This should include correcting punctuation, unnecessary capitalization and addressing spacing issues. For example, in the sentence 'cover the entire country before declaring it free of Onchocerciasis By close of 2017, three countries -Republic of Venezuela, Uganda, and Sudan', there are a few areas needing attention:  1) A period is needed after 'onchocerciasis'. 2) 'By' should be preceded by 'the'. 3) A space is required after the hyphen (and perhaps another hyphen should be added after Sudan). 4) The term 'Republic' should be pluralised to 'Republics of' to correctly refer to the countries mentioned. | Thank you very much for this comment. This has been addressed on page four | See page 4 |
| Moreover, for scientific writing, it's advisable to spell out numbers from one to ten. For instance, 'As of 2023, the country still has 7 districts in PTS, while 5 border districts are under...' could be more formally written as 'As of 2023, the country still has seven districts in PTS, while five border districts are under...' | Thank you for this comment. This has been addressed | See page 4 |
| Summary and General Comments |  |  |
| The study by Khainza et al., titled “Community Involvement in Post-elimination Surveillance in Bududa District, Eastern Uganda: a cross-sectional study,” is a significant contribution to our understanding of onchocerciasis post-elimination surveillance (PES) in Eastern Uganda. Its emphasis on community involvement during the post-elimination phase is particularly timely and relevant, as many African nations are nearing the goal of onchocerciasis elimination. The findings, which point to a low level of community involvement in PES activities, offer critical insights that could inform future strategies in similar contexts.  The study underscores the importance of increasing community involvement in PES activities, ideally incorporating Community Drug Distributors (CDDs) and other professionals who have been involved in prior onchocerciasis elimination efforts. Establishing an effective onchocerciasis referral system, complete with necessary drugs and diagnostic equipment, is crucial for sustaining the gains made in onchocerciasis control and elimination. | Thank you very much for this comment. |  |

Thank you once again for your commitment to the review process, and I look forward to your further guidance.

Sincerely,

Khainza Tabitha Annet

Buistema University

+256772650124
