# Supplementary material for: Community Involvement in Onchocerciasis Post-elimination Surveillance in Bududa District, Eastern Uganda: A cross-sectional study
Source: PLoS Negl Trop Dis. 2024 Jul 16;18(7):e0012270. doi: 10.1371/journal.pntd.0012270 (PMC11251607; doi:10.1371/journal.pntd.0012270)
Supplement: Attachment — Submitted filename: Point by Point Response letter_Final.docx [file pntd.0012270.s003.docx]

**Subject: Revised Manuscript Submission - "Community Involvement in Post-elimination Surveillance in Bududa District, Eastern Uganda: A Cross-Sectional Study" to Neglected Tropical Diseases**

Dear Matthew C Freeman,

I extend my sincere gratitude for affording me the opportunity to submit another revised draft of my manuscript, titled "Community Involvement in Post-elimination Surveillance in Bududa District, Eastern Uganda: A Cross-Sectional Study," to Neglected Tropical Diseases. I want to express my sincere appreciation for the time and effort you have devoted to providing enhancing the quality of this work. I am particularly thankful to the reviewers for their insightful comments, which have strengthened the quality of my paper. I am thankful for your dedication on the review of our manuscript. Enclosed is our detailed point-by-point response to each comment for your review.

| **Reviewer’s comment** | **Response to comment** | **Page numbers** |
| --- | --- | --- |
| Reviewer #1 | | |
| **Methods** |  |  |
| No comments |  |  |
| **Results** |  |  |
| Some of the tables are overlapping, which is incorrect. I suggest that if a table cannot fit into one page, the authors should break that into sections maintaining the table and columns headings.  • I suggest that the statistical tool/s employed in the analysis of the results should be spelt out clearly under ‘Data analysis’ statement.  • The description of the results under Implementation of Onchocerciasis PES Activities’, is not found in the table. For instance, where can 181 (42.9%) be found in the table. Also, when describing /interpreting the results, provide reference to the Table or Figures where they can be found | The statistical tools have also been added under the data analysis section  Thank you for noting the irregularity with the table. This has been corrected in the write up under table 5. Line number 356 |  |
| **Conclusion** |  |  |
| No comment |  |  |
| **Editorial and Data Presentation Modifications** |  |  |
| I suggest the authors take into consideration the recommendations made to enhance the clarity of the manuscript to the reader. | Noted with thanks |  |
| **Summary and General Comments** |  |  |
| The manuscript still needs some further work: 1.The authors failed to provide line numbers as recommended making it difficult to provide review comments to specifics in the manuscript. | The line numbers have now been provided on the manuscript |  |
| 2. The manuscript contains incorrect sentence constructions. The authors failed to provide full-stop at the end of some sentences, no ‘space’ to separate some words and sentences in the manuscript. | Thank you for this comment. This has also now been harmonized throughout the entire document |  |
| 3. The authors have also failed to provide information on the authors’ contributions as recommended. I suggest the authors provide this information after the acknowledgement statement. | Thank you for this insight. This has now been introduced before the acknowledgement page. |  |
| 4.Please for the use of abbreviations in the write-up, kindly identify the first appearance of such words in the manuscript, write them in full and the abbreviated forms in brackets after which the abbreviated forms alone can subsequently be used in the write-up. Please the authors are encouraged to correct the anomalies in the manuscript. | Thank you for these comments. This has been also adopted, with changes coming in areas like method section “focus group discussions (FGDs)” |  |
| 5.The in-text citation is incorrect. It is square bracket not parenthesis. I suggest the authors correct it. | The recommended squared bracket has also no been introduced. See all the in-text citations |  |
| 6. The bibliographies have not been edited to suit the journal’s style as recommended. The authors should kindly follow the journal’s submission guidelines to format each of their bibliographies according to source | The bibliography has also now been edited to suit the PLOS NTD style. |  |
| Reviewer #2 | | |
| Study Design: Regarding "This was a cross sectional study that utilized both quantitative and qualitative data collection methods. Perhaps a more in-depht information could be given by mentioning the semi-structured questionnaire for the quantitative part and focused group discussions/kei informant interviews for the qualitative part.  As mentioned before, the mixed-methods study design is beneficial. Moreover, the ethics approval and consent is now clearly described. The sample size is clearly described and produced, although with a typo. I believe the authors mean 50.0% instead of 0,50% of expected community involvement. As one would only need 20 participants for a proportion of 0.50%.Method | Thank you very much for this comment. We have provided more in-depth information on the data collection tools separately. For instance, for quantitative aspect, we specified the tool being semi structured and for qualitative we also specified the tool being KIIs and FGDs.  Regarding the sample, the use of 0.50% was a typos error, which has been corrected in line number 164.  Additionally, we have also highlighted this under study design, line numbers 131 and 132. | See page 7, line number 192 for quantitative and qualitative page 10, line number 247. |
| **Results** |  |  |
| The authors now better described the regression section.  In the discussions, a more nuanced approach may be needed, particularly to better differentiate it from the results sections, including adding more references.  Referral for oncho still not practiced in this PES area, and this is indeed an important issue that needs to be addressed and prevented. It was mentioned that there were referred suspected oncho cases. Is it known about their whereabouts, that is, if the diagnosis was confirmed and, if so, treated? As mentioned, it is indeed important to have ivermectin available and the doctors aware of onchocerciasis and its clinical manifestations. | Thank you very much  Much as the suspected cases were clinically referred no diagnosis was done due to lack of skills and materials. No treatment was done because there was no confirmed diagnosis. |  |
| **Conclusion** |  |  |
| Limitations of the study are now mentioned.  The conclusions are supported by the data presented in the manuscript and can benefit the knowledge on the topic of PES of onchocerciasis. | Thank you for this comment |  |
| **Editorial and Data Presentation Modifications** |  |  |
| Introduction:  1) Break down long paragraphs (e.g., starting with "In 2017, the Global Burden of Disease study estimated..."). | Thank you for this suggestion. This statement has been broken into two parts that reads, “In 2017, the Global Burden of Disease study estimated a worldwide total of approximately 20.9 million prevalent O. volvulus infections. Among these cases, 14.6 million individuals experienced skin diseases, and 1.15 million suffered from vision loss” | See line numbers 59,60,61. |
| 2) Mention "once or twice a year" for Ivermectin use in Africa compared to the Americas. | This has been included in line numbers 69 and 70. | Page 3 |
| 3) Avoid portraying temephos as entirely "chemically safe." Acknowledge potential environmental concerns | This comment has also been adopted and caution put on the use of temephos | See line number 74,75 and 76 |
| 4) "The positive outcomes observed in these affected regions can be attributed to strong collaborations among diverse stakeholders, including international donors, the WHO, governments, NGOs and communities (3){Gebrezgabiher, 2019 #1092}." I would say "partially attributed", as in the Americas there were also other drivers of successful onchcoerciasis elimination, such as less competent vectors | This has been adopted as modified to consider partially as recommended. See line number 109. | Page 4 |
| 5) In "As of 2023, the country still has 7 districts in Post-Treatment Surveillance (PTS)", consider using "had" instead of "has" for districts in past PTS. | Thank you for this insight. This grammatical error has been rectified in line 115. | Page 5 |
| 4) Specify annual or biannual reasons for Ivermectin distribution in Uganda. | Thank you very much for this comment. This strategy for Uganda, was guided by recommendations from the World Health Organization (WHO) and other relevant health authorities. These guidelines typically advocate for annual or biannual treatment depending on the endemicity and transmission dynamics of onchocerciasis in a given area | See page 5, line numbers 114 to 117 |
| Overall English Language: The article requires substantial improvement, particularly in the introduction and, to a lesser extent, in the methodology. Consider: | Thank you very much for this comment. The language has been improved through the assistance of a language expert |  |
| 1) Avoiding capitalization of words like "onchocerciasis," "ivermectin,", "post-", "annual", "country", etc. | This recommendation has been incorporated throughout the entire document |  |
| 2) Writing numbers one to ten in full text (e.g., "one to ten"). | This has been adopted throughout the document. For example, 7 changed to “seven” under study setting | See line number 156 |
| 3) Addressing inconsistencies in text size and spacing. | This has been addressed throughout the document. For instance, the word Onchocerciasis has been changed to “onchocerciasis” | See line number 162 |
| 4) Correcting punctuation (e.g., lack or excess of commas). | This has been addressing across the entire document |  |
| Abstract: Define AOR before using the abbreviation and mention the methodology used to calculate AOR (e.g. " logistic regression to assess factors associated with involvement"). | This has been defined very clearly now in the abstract and other places in the result. |  |
| Other points: 1) Ensure proper punctuation (e.g., "e.g. parasitic worm Onchocerca volvulus (O. volvulus) (1) (2)T his"). | The “punctuation” has now been affected as recommended. |  |
| 2)Avoid paraphrasing established terms (e.g., "ailment" instead of "disease"). | This has now been rectified by changing “ailment” to disease |  |
| 3)Standardize spacing throughout the manuscript. | The spacing of the manuscript has been harmonized to 2.0 line spacing |  |
| 6) In "fisher exact where " suggest to mention it as "Fisher exact test". Great that the logistic regression is now mentioned in the data analysis plan | This has been corrected as recommended |  |
| 7) Add "to" after "communities implement sound" in the methods section. | The word to has been added as recommended on line 126 |  |
| 8) Define Mount Elgon at first mention (excluding abstract), instead of Mt.. Provide a reference for its baseline prevalence if available. | This has been defined in line 143 under study settings. |  |
| ) Explain DDT abbreviation on first mention in the text (excluding abstract). | The abbreviation DDT has been defined in line 148, under study settings |  |
| 10) In  "Annual  mass treatment with ivermectin changed to two times per year along with vector elimination  in 2007", clarify if vector control strategies were implemented in 2007, or if vector elimination was achieved in 2007. | Thank you for this comment. We clarified this in line 150. The vector control was relaunched in 2007 after its successful use during the elimination of the Victoria focus in 1970. |  |
| 11) In "and had the highest prevalence rate (68.2% for Bushika and 56.3 % for Bukigai).  " I would suggest mentioning "at baseline" or "before control interventions". Also, a reference would be beneficial. | Thank you for this comment. We have corrected this to reflect the mean onchocerciasis prevalence before control intervention, of 80%. However, the whole of Bukigai and Bushika were endemic at baseline. | See line numbers 147 and 184 |
| 12) Define FGDs and KIIs on first mention in the article (excluding abstract). | This has been defined in the document under qualitative data analysis section and study design section. |  |
| 13) Improve spacing and punctuation in results sections. Such as "more than half 216/422(51 .2%) were females " perhaps as "more than halve (216/422, 51.2%) were females". | This has been addressed as recommended |  |
| 14) In the result, it could be beneficial to have a background on the blackfly control history, such as how much was the ABR (bites per person per year) before control, how much did it decrease during control, and did it recover afterwards and how? (if info available) to link with the results on sights of blackflies of the current studies. | Thanks to this comment, however, according to our literature search, the information on the ABR (bites per person per year) before control was not available. |  |
| 15) In the results and discussion, What is it meant by "district support"? Is it financial, logistical, supervision...? Perhaps define it in the methods or results. | The district support was mainly financial and supervision. | See Line numbers 388 and 389 |
| 16) In the discussion: "environment in a week [AOR 8, 95% CI= (1.5, 42.5)], in one month" should perhaps be "environment in the previous week to the survey [AOR 8, 95% CI= (1.5, 42.5)], in the previous month to the survey" ? | This has been corrected to match the suggestion proposed under the discussion section | See page 29 |
| **Summary and General Comments** |  |  |
| This study offers valuable insights into community involvement in PES for onchocerciasis elimination. The mixed-methods approach is a strength. The low community involvement identified highlights a potential gap between program focus on elimination and neglecting elimination surveillance activities. Additionally, logistical challenges in reaching remote areas may need to be addressed. The study's generalizability is limited by the cross-sectional design and single district focus. The findings on factors associated with involvement (district support, black fly sightings, volunteer experience) and identified challenges (funding, motivation, program sustainability, drug availability) are significant for improving community involvement in PES programs. The authors should strengthen the discussion section with more references to support their claims. Consider including page and line numbers for future revisions (facilitates commenting).  Encouragingly, the authors have addressed most of the concerns raised in a previous review. With further revisions to strengthen the presentation, discussion section and overall language clarity, this study has the potential to be a valuable contribution to the literature on community involvement in onchocerciasis elimination programs. | Thank you very much for this encouraging and brilliant comment |  |

Thank you once again for your commitment to the review process, and I look forward to your further guidance.

Sincerely,

Khainza Tabitha Annet

Buistema University

+256772650124
